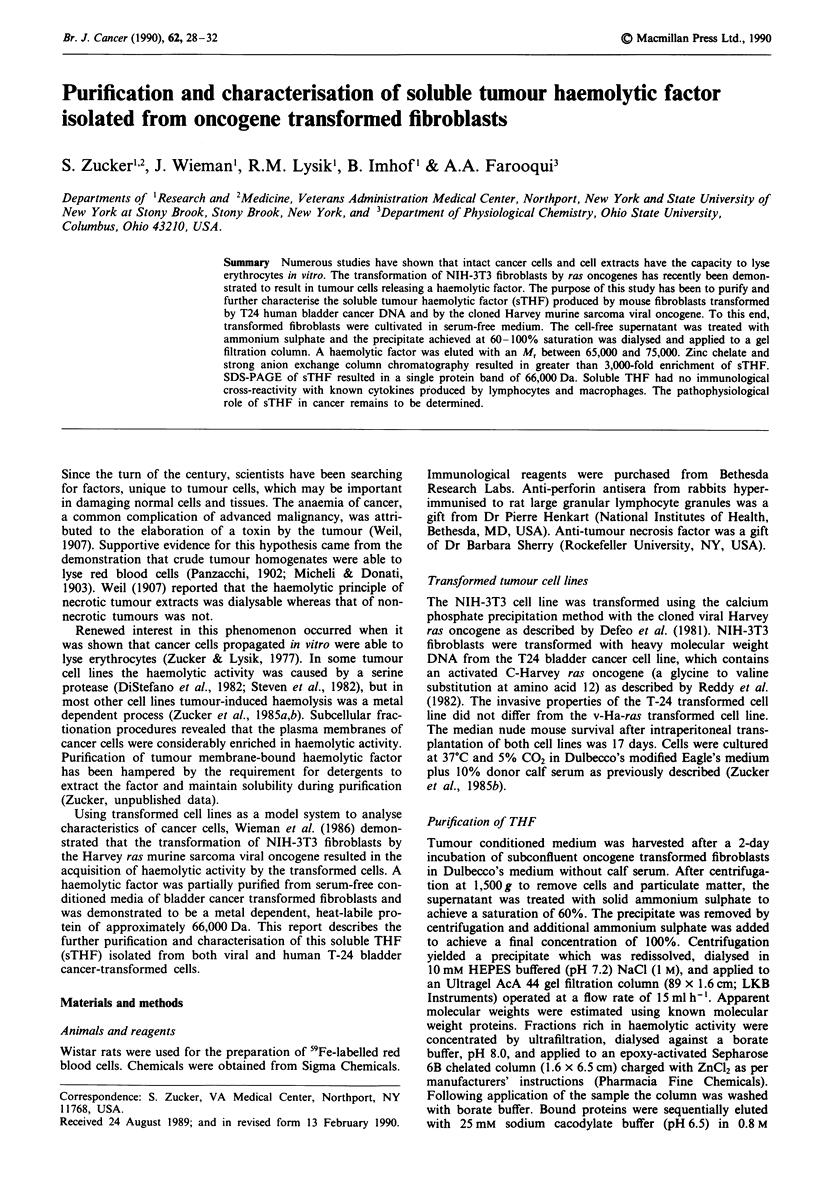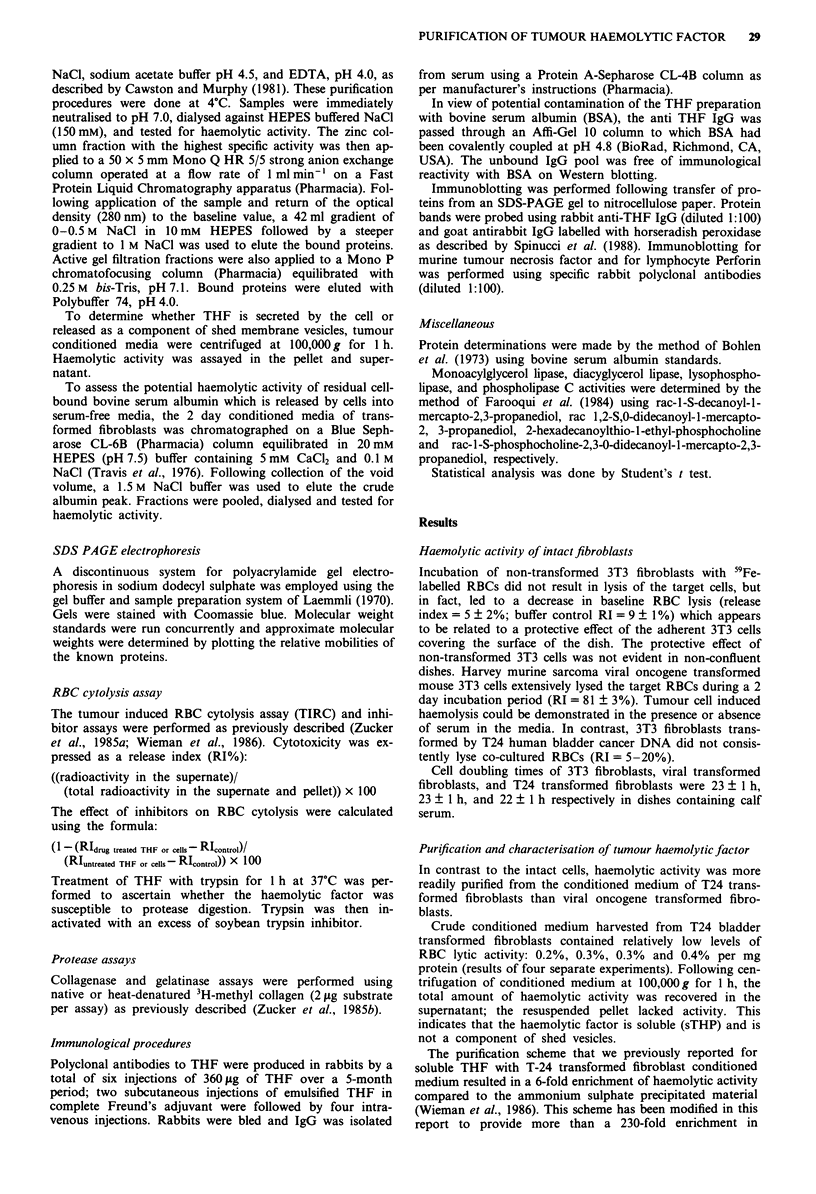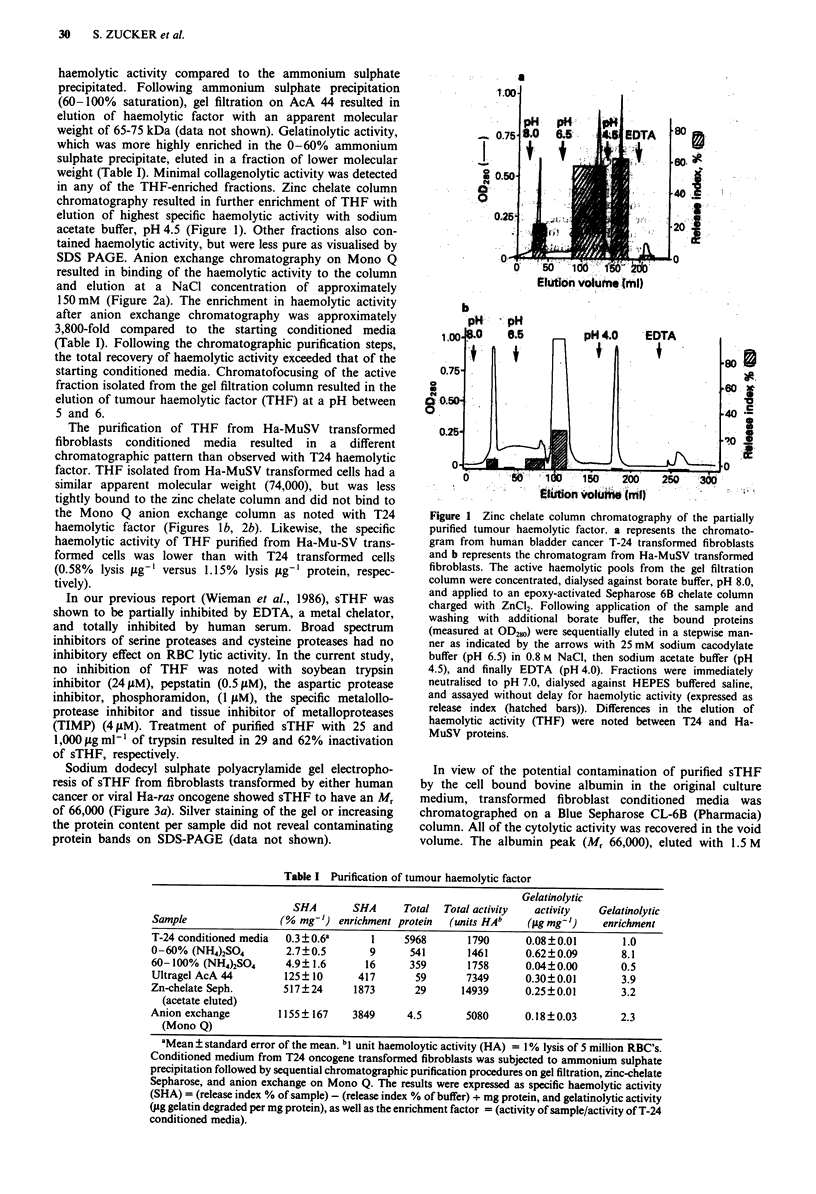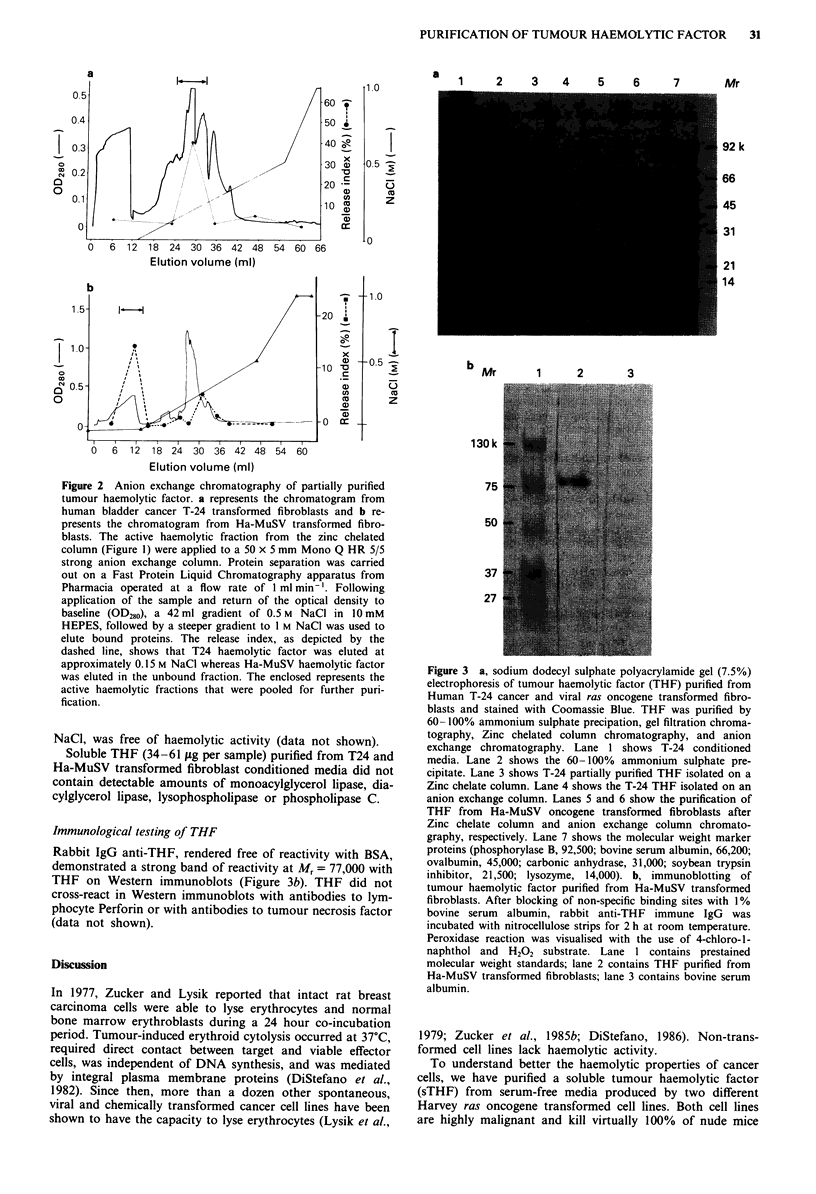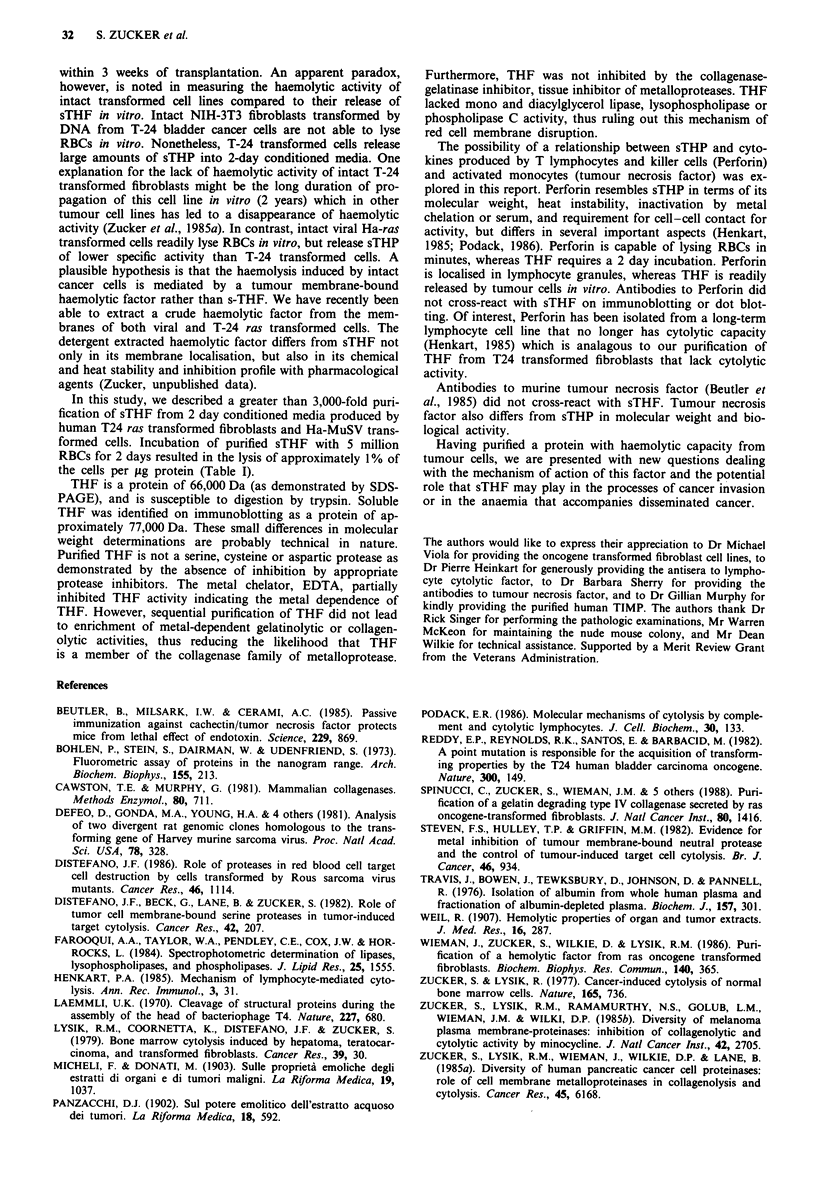# Purification and characterisation of soluble tumour haemolytic factor isolated from oncogene transformed fibroblasts.

**DOI:** 10.1038/bjc.1990.223

**Published:** 1990-07

**Authors:** S. Zucker, J. Wieman, R. M. Lysik, B. Imhof, A. A. Farooqui

**Affiliations:** Department of Research, Veterans Administration Medical Center, Northport, New York 11768.

## Abstract

**Images:**


Br. J. Cancer (1990), 62, 28-32
© Macmillan Press Ltd., 1990

### Purification and characterisation of soluble tumour haemolytic factor isolated from oncogene transformed fibroblasts

S. Zucker[1,2], J. Wieman[1], R.M. Lysik[1], B. Imhof[1] & A.A. Farooqui[3]

*Departments of [1]Research and [2]Medicine, Veterans Administration Medical Center, Northport, New York and State University of New York at Stony Brook, Stony Brook, New York, and [3]Department of Physiological Chemistry, Ohio State University, Columbus, Ohio 43210, USA.*

**Summary** Numerous studies have shown that intact cancer cells and cell extracts have the capacity to lyse erythrocytes *in vitro*. The transformation of NIH-3T3 fibroblasts by *ras* oncogenes has recently been demonstrated to result in tumour cells releasing a haemolytic factor. The purpose of this study has been to purify and further characterise the soluble tumour haemolytic factor (sTHF) produced by mouse fibroblasts transformed by T24 human bladder cancer DNA and by the cloned Harvey murine sarcoma viral oncogene. To this end, transformed fibroblasts were cultivated in serum-free medium. The cell-free supernatant was treated with ammonium sulphate and the precipitate achieved at 60-100% saturation was dialysed and applied to a gel filtration column. A haemolytic factor was eluted with an $M_r$ between 65,000 and 75,000. Zinc chelate and strong anion exchange column chromatography resulted in greater than 3,000-fold enrichment of sTHF. SDS-PAGE of sTHF resulted in a single protein band of 66,000 Da. Soluble THF had no immunological cross-reactivity with known cytokines produced by lymphocytes and macrophages. The pathophysiological role of sTHF in cancer remains to be determined.

Since the turn of the century, scientists have been searching for factors, unique to tumour cells, which may be important in damaging normal cells and tissues. The anaemia of cancer, a common complication of advanced malignancy, was attributed to the elaboration of a toxin by the tumour (Weil, 1907). Supportive evidence for this hypothesis came from the demonstration that crude tumour homogenates were able to lyse red blood cells (Panzacchi, 1902; Micheli & Donati, 1903). Weil (1907) reported that the haemolytic principle of necrotic tumour extracts was dialysable whereas that of non-necrotic tumours was not.

Renewed interest in this phenomenon occurred when it was shown that cancer cells propagated *in vitro* were able to lyse erythrocytes (Zucker & Lysik, 1977). In some tumour cell lines the haemolytic activity was caused by a serine protease (DiStefano *et al.*, 1982; Steven *et al.*, 1982), but in most other cell lines tumour-induced haemolysis was a metal dependent process (Zucker *et al.*, 1985*a*,*b*). Subcellular fractionation procedures revealed that the plasma membranes of cancer cells were considerably enriched in haemolytic activity. Purification of tumour membrane-bound haemolytic factor has been hampered by the requirement for detergents to extract the factor and maintain solubility during purification (Zucker, unpublished data).

Using transformed cell lines as a model system to analyse characteristics of cancer cells, Wieman *et al.* (1986) demonstrated that the transformation of NIH-3T3 fibroblasts by the Harvey *ras* murine sarcoma viral oncogene resulted in the acquisition of haemolytic activity by the transformed cells. A haemolytic factor was partially purified from serum-free conditioned media of bladder cancer transformed fibroblasts and was demonstrated to be a metal dependent, heat-labile protein of approximately 66,000 Da. This report describes the further purification and characterisation of this soluble THF (sTHF) isolated from both viral and human T-24 bladder cancer-transformed cells.

### Materials and methods

### *Animals and reagents*

Wistar rats were used for the preparation of $^{59}$Fe-labelled red blood cells. Chemicals were obtained from Sigma Chemicals. Immunological reagents were purchased from Bethesda Research Labs. Anti-perforin antisera from rabbits hyperimmunised to rat large granular lymphocyte granules was a gift from Dr Pierre Henkart (National Institutes of Health, Bethesda, MD, USA). Anti-tumour necrosis factor was a gift of Dr Barbara Sherry (Rockefeller University, NY, USA).

### *Transformed tumour cell lines*

The NIH-3T3 cell line was transformed using the calcium phosphate precipitation method with the cloned viral Harvey *ras* oncogene as described by Defeo *et al.* (1981). NIH-3T3 fibroblasts were transformed with heavy molecular weight DNA from the T24 bladder cancer cell line, which contains an activated C-Harvey *ras* oncogene (a glycine to valine substitution at amino acid 12) as described by Reddy *et al.* (1982). The invasive properties of the T-24 transformed cell line did not differ from the v-Ha-*ras* transformed cell line. The median nude mouse survival after intraperitoneal transplantation of both cell lines was 17 days. Cells were cultured at 37°C and 5% $CO_2$ in Dulbecco's modified Eagle's medium plus 10% donor calf serum as previously described (Zucker *et al.*, 1985*b*).

### *Purification of THF*

Tumour conditioned medium was harvested after a 2-day incubation of subconfluent oncogene transformed fibroblasts in Dulbecco's medium without calf serum. After centrifugation at 1,500 *g* to remove cells and particulate matter, the supernatant was treated with solid ammonium sulphate to achieve a saturation of 60%. The precipitate was removed by centrifugation and additional ammonium sulphate was added to achieve a final concentration of 100%. Centrifugation yielded a precipitate which was redissolved, dialysed in 10 mM HEPES buffered (pH 7.2) NaCl (1 M), and applied to an Ultragel AcA 44 gel filtration column (89 × 1.6 cm; LKB Instruments) operated at a flow rate of 15 ml h$^{-1}$. Apparent molecular weights were estimated using known molecular weight proteins. Fractions rich in haemolytic activity were concentrated by ultrafiltration, dialysed against a borate buffer, pH 8.0, and applied to an epoxy-activated Sepharose 6B chelated column (1.6 × 6.5 cm) charged with $ZnCl_2$ as per manufacturers' instructions (Pharmacia Fine Chemicals). Following application of the sample the column was washed with borate buffer. Bound proteins were sequentially eluted with 25 mM sodium cacodylate buffer (pH 6.5) in 0.8 M

Correspondence: S. Zucker, VA Medical Center, Northport, NY 11768, USA.
Received 24 August 1989; and in revised form 13 February 1990.

NaCl, sodium acetate buffer pH 4.5, and EDTA, pH 4.0, as described by Cawston and Murphy (1981). These purification procedures were done at 4°C. Samples were immediately neutralised to pH 7.0, dialysed against HEPES buffered NaCl (150 mM), and tested for haemolytic activity. The zinc column fraction with the highest specific activity was then applied to a 50 × 5 mm Mono Q HR 5/5 strong anion exchange column operated at a flow rate of 1 ml $min^{-1}$ on a Fast Protein Liquid Chromatography apparatus (Pharmacia). Following application of the sample and return of the optical density (280 nm) to the baseline value, a 42 ml gradient of 0–0.5 M NaCl in 10 mM HEPES followed by a steeper gradient to 1 M NaCl was used to elute the bound proteins. Active gel filtration fractions were also applied to a Mono P chromatofocusing column (Pharmacia) equilibrated with 0.25 M *bis*-Tris, pH 7.1. Bound proteins were eluted with Polybuffer 74, pH 4.0.

To determine whether THF is secreted by the cell or released as a component of shed membrane vesicles, tumour conditioned media were centrifuged at 100,000 *g* for 1 h. Haemolytic activity was assayed in the pellet and supernatant.

To assess the potential haemolytic activity of residual cell-bound bovine serum albumin which is released by cells into serum-free media, the 2 day conditioned media of transformed fibroblasts was chromatographed on a Blue Sepharose CL-6B (Pharmacia) column equilibrated in 20 mM HEPES (pH 7.5) buffer containing 5 mM $CaCl_2$ and 0.1 M NaCl (Travis *et al.*, 1976). Following collection of the void volume, a 1.5 M NaCl buffer was used to elute the crude albumin peak. Fractions were pooled, dialysed and tested for haemolytic activity.

### *SDS PAGE electrophoresis*

A discontinuous system for polyacrylamide gel electrophoresis in sodium dodecyl sulphate was employed using the gel buffer and sample preparation system of Laemmli (1970). Gels were stained with Coomassie blue. Molecular weight standards were run concurrently and approximate molecular weights were determined by plotting the relative mobilities of the known proteins.

### *RBC cytolysis assay*

The tumour induced RBC cytolysis assay (TIRC) and inhibitor assays were performed as previously described (Zucker *et al.*, 1985*a*; Wieman *et al.*, 1986). Cytotoxicity was expressed as a release index (RI%):

((radioactivity in the supernate)/
(total radioactivity in the supernate and pellet)) × 100

The effect of inhibitors on RBC cytolysis were calculated using the formula:

$$(1 - (RI_{\text{drug treated THF or cells}} - RI_{\text{control}}) / (RI_{\text{untreated THF or cells}} - RI_{\text{control}})) \times 100$$

Treatment of THF with trypsin for 1 h at 37°C was performed to ascertain whether the haemolytic factor was susceptible to protease digestion. Trypsin was then inactivated with an excess of soybean trypsin inhibitor.

### *Protease assays*

Collagenase and gelatinase assays were performed using native or heat-denatured $^3$H-methyl collagen (2 μg substrate per assay) as previously described (Zucker *et al.*, 1985*b*).

### *Immunological procedures*

Polyclonal antibodies to THF were produced in rabbits by a total of six injections of 360 μg of THF over a 5-month period; two subcutaneous injections of emulsified THF in complete Freund's adjuvant were followed by four intravenous injections. Rabbits were bled and IgG was isolated from serum using a Protein A-Sepharose CL-4B column as per manufacturer's instructions (Pharmacia).

In view of potential contamination of the THF preparation with bovine serum albumin (BSA), the anti THF IgG was passed through an Affi-Gel 10 column to which BSA had been covalently coupled at pH 4.8 (BioRad, Richmond, CA, USA). The unbound IgG pool was free of immunological reactivity with BSA on Western blotting.

Immunoblotting was performed following transfer of proteins from an SDS-PAGE gel to nitrocellulose paper. Protein bands were probed using rabbit anti-THF IgG (diluted 1:100) and goat antirabbit IgG labelled with horseradish peroxidase as described by Spinucci *et al.* (1988). Immunoblotting for murine tumour necrosis factor and for lymphocyte Perforin was performed using specific rabbit polyclonal antibodies (diluted 1:100).

### *Miscellaneous*

Protein determinations were made by the method of Bohlen *et al.* (1973) using bovine serum albumin standards.

Monoacylglycerol lipase, diacyglycerol lipase, lysophospholipase, and phospholipase C activities were determined by the method of Farooqui *et al.* (1984) using rac-1-S-decanoyl-1-mercapto-2,3-propanediol, rac 1,2-S,0-didecanoyl-1-mercapto-2, 3-propanediol, 2-hexadecanoylthio-1-ethyl-phosphocholine and rac-1-S-phosphocholine-2,3-0-didecanoyl-1-mercapto-2,3-propanediol, respectively.

Statistical analysis was done by Student's *t* test.

### Results

### *Haemolytic activity of intact fibroblasts*

Incubation of non-transformed 3T3 fibroblasts with $^{59}$Fe-labelled RBCs did not result in lysis of the target cells, but in fact, led to a decrease in baseline RBC lysis (release index = 5 ± 2%; buffer control RI = 9 ± 1%) which appears to be related to a protective effect of the adherent 3T3 cells covering the surface of the dish. The protective effect of non-transformed 3T3 cells was not evident in non-confluent dishes. Harvey murine sarcoma viral oncogene transformed mouse 3T3 cells extensively lysed the target RBCs during a 2 day incubation period (RI = 81 ± 3%). Tumour cell induced haemolysis could be demonstrated in the presence or absence of serum in the media. In contrast, 3T3 fibroblasts transformed by T24 human bladder cancer DNA did not consistently lyse co-cultured RBCs (RI = 5–20%).

Cell doubling times of 3T3 fibroblasts, viral transformed fibroblasts, and T24 transformed fibroblasts were 23 ± 1 h, 23 ± 1 h, and 22 ± 1 h respectively in dishes containing calf serum.

### *Purification and characterisation of tumour haemolytic factor*

In contrast to the intact cells, haemolytic activity was more readily purified from the conditioned medium of T24 transformed fibroblasts than viral oncogene transformed fibroblasts.

Crude conditioned medium harvested from T24 bladder transformed fibroblasts contained relatively low levels of RBC lytic activity: 0.2%, 0.3%, 0.3% and 0.4% per mg protein (results of four separate experiments). Following centrifugation of conditioned medium at 100,000 *g* for 1 h, the total amount of haemolytic activity was recovered in the supernatant; the resuspended pellet lacked activity. This indicates that the haemolytic factor is soluble (sTHP) and is not a component of shed vesicles.

The purification scheme that we previously reported for soluble THF with T-24 transformed fibroblast conditioned medium resulted in a 6-fold enrichment of haemolytic activity compared to the ammonium sulphate precipitated material (Wieman *et al.*, 1986). This scheme has been modified in this report to provide more than a 230-fold enrichment in haemolytic activity compared to the ammonium sulphate precipitated. Following ammonium sulphate precipitation (60–100% saturation), gel filtration on AcA 44 resulted in elution of haemolytic factor with an apparent molecular weight of 65-75 kDa (data not shown). Gelatinolytic activity, which was more highly enriched in the 0–60% ammonium sulphate precipitate, eluted in a fraction of lower molecular weight (Table I). Minimal collagenolytic activity was detected in any of the THF-enriched fractions. Zinc chelate column chromatography resulted in further enrichment of THF with elution of highest specific haemolytic activity with sodium acetate buffer, pH 4.5 (Figure 1). Other fractions also contained haemolytic activity, but were less pure as visualised by SDS PAGE. Anion exchange chromatography on Mono Q resulted in binding of the haemolytic activity to the column and elution at a NaCl concentration of approximately 150 mM (Figure 2a). The enrichment in haemolytic activity after anion exchange chromatography was approximately 3,800-fold compared to the starting conditioned media (Table I). Following the chromatographic purification steps, the total recovery of haemolytic activity exceeded that of the starting conditioned media. Chromatofocusing of the active fraction isolated from the gel filtration column resulted in the elution of tumour haemolytic factor (THF) at a pH between 5 and 6.

The purification of THF from Ha-MuSV transformed fibroblasts conditioned media resulted in a different chromatographic pattern than observed with T24 haemolytic factor. THF isolated from Ha-MuSV transformed cells had a similar apparent molecular weight (74,000), but was less tightly bound to the zinc chelate column and did not bind to the Mono Q anion exchange column as noted with T24 haemolytic factor (Figures 1b, 2b). Likewise, the specific haemolytic activity of THF purified from Ha-Mu-SV transformed cells was lower than with T24 transformed cells (0.58% lysis $\mu g^{-1}$ versus 1.15% lysis $\mu g^{-1}$ protein, respectively).

In our previous report (Wieman et al., 1986), sTHF was shown to be partially inhibited by EDTA, a metal chelator, and totally inhibited by human serum. Broad spectrum inhibitors of serine proteases and cysteine proteases had no inhibitory effect on RBC lytic activity. In the current study, no inhibition of THF was noted with soybean trypsin inhibitor (24 μM), pepstatin (0.5 μM), the aspartic protease inhibitor, phosphoramidon, (1 μM), the specific metalolloprotease inhibitor and tissue inhibitor of metalloproteases (TIMP) (4 μM). Treatment of purified sTHF with 25 and 1,000 $\mu g\ ml^{-1}$ of trypsin resulted in 29 and 62% inactivation of sTHF, respectively.

Sodium dodecyl sulphate polyacrylamide gel electrophoresis of sTHF from fibroblasts transformed by either human cancer or viral Ha-*ras* oncogene showed sTHF to have an $M_r$ of 66,000 (Figure 3a). Silver staining of the gel or increasing the protein content per sample did not reveal contaminating protein bands on SDS-PAGE (data not shown).

**Figure 1** Zinc chelate column chromatography of the partially purified tumour haemolytic factor. **a** represents the chromatogram from human bladder cancer T-24 transformed fibroblasts and **b** represents the chromatogram from Ha-MuSV transformed fibroblasts. The active haemolytic pools from the gel filtration column were concentrated, dialysed against borate buffer, pH 8.0, and applied to an epoxy-activated Sepharose 6B chelate column charged with $ZnCl_2$. Following application of the sample and washing with additional borate buffer, the bound proteins (measured at $OD_{280}$) were sequentially eluted in a stepwise manner as indicated by the arrows with 25 mM sodium cacodylate buffer (pH 6.5) in 0.8 M NaCl, then sodium acetate buffer (pH 4.5), and finally EDTA (pH 4.0). Fractions were immediately neutralised to pH 7.0, dialysed against HEPES buffered saline, and assayed without delay for haemolytic activity (expressed as release index (hatched bars)). Differences in the elution of haemolytic activity (THF) were noted between T24 and Ha-MuSV proteins.

In view of the potential contamination of purified sTHF by the cell bound bovine albumin in the original culture medium, transformed fibroblast conditioned media was chromatographed on a Blue Sepharose CL-6B (Pharmacia) column. All of the cytolytic activity was recovered in the void volume. The albumin peak ($M_r$ 66,000), eluted with 1.5 M

**Table I** Purification of tumour haemolytic factor

| *Sample* | *SHA (% $mg^{-1}$)* | *SHA enrichment* | *Total protein* | *Total activity (units HA[b]* | *Gelatinolytic activity ($\mu g\ mg^{-1}$)* | *Gelatinolytic enrichment* |
|---|---|---|---|---|---|---|
| T-24 conditioned media | 0.3±0.6[a] | 1 | 5968 | 1790 | 0.08±0.01 | 1.0 |
| 0–60% $(NH_4)_2SO_4$ | 2.7±0.5 | 9 | 541 | 1461 | 0.62±0.09 | 8.1 |
| 60–100% $(NH_4)_2SO_4$ | 4.9±1.6 | 16 | 359 | 1758 | 0.04±0.00 | 0.5 |
| Ultragel AcA 44 | 125±10 | 417 | 59 | 7349 | 0.30±0.01 | 3.9 |
| Zn-chelate Seph. (acetate eluted) | 517±24 | 1873 | 29 | 14939 | 0.25±0.01 | 3.2 |
| Anion exchange (Mono Q) | 1155±167 | 3849 | 4.5 | 5080 | 0.18±0.03 | 2.3 |

[a]Mean ± standard error of the mean. [b]1 unit haemoloytic activity (HA) = 1% lysis of 5 million RBC's. Conditioned medium from T24 oncogene transformed fibroblasts was subjected to ammonium sulphate precipitation followed by sequential chromatographic purification procedures on gel filtration, zinc-chelate Sepharose, and anion exchange on Mono Q. The results were expressed as specific haemolytic activity (SHA) = (release index % of sample) − (release index % of buffer) ÷ mg protein, and gelatinolytic activity (μg gelatin degraded per mg protein), as well as the enrichment factor = (activity of sample/activity of T-24 conditioned media).

**Figure 2** Anion exchange chromatography of partially purified tumour haemolytic factor. **a** represents the chromatogram from human bladder cancer T-24 transformed fibroblasts and **b** represents the chromatogram from Ha-MuSV transformed fibroblasts. The active haemolytic fraction from the zinc chelated column (Figure 1) were applied to a 50 × 5 mm Mono Q HR 5/5 strong anion exchange column. Protein separation was carried out on a Fast Protein Liquid Chromatography apparatus from Pharmacia operated at a flow rate of 1 ml $min^{-1}$. Following application of the sample and return of the optical density to baseline ($OD_{280}$), a 42 ml gradient of 0.5 M NaCl in 10 mM HEPES, followed by a steeper gradient to 1 M NaCl was used to elute bound proteins. The release index, as depicted by the dashed line, shows that T24 haemolytic factor was eluted at approximately 0.15 M NaCl whereas Ha-MuSV haemolytic factor was eluted in the unbound fraction. The enclosed represents the active haemolytic fractions that were pooled for further purification.

**Figure 3** **a**, sodium dodecyl sulphate polyacrylamide gel (7.5%) electrophoresis of tumour haemolytic factor (THF) purified from Human T-24 cancer and viral *ras* oncogene transformed fibroblasts and stained with Coomassie Blue. THF was purified by 60–100% ammonium sulphate precipation, gel filtration chromatography, Zinc chelated column chromatography, and anion exchange chromatography. Lane 1 shows T-24 conditioned media. Lane 2 shows the 60–100% ammonium sulphate precipitate. Lane 3 shows T-24 partially purified THF isolated on a Zinc chelate column. Lane 4 shows the T-24 THF isolated on an anion exchange column. Lanes 5 and 6 show the purification of THF from Ha-MuSV oncogene transformed fibroblasts after Zinc chelate column and anion exchange column chromatography, respectively. Lane 7 shows the molecular weight marker proteins (phosphorylase B, 92,500; bovine serum albumin, 66,200; ovalbumin, 45,000; carbonic anhydrase, 31,000; soybean trypsin inhibitor, 21,500; lysozyme, 14,000). **b**, immunoblotting of tumour haemolytic factor purified from Ha-MuSV transformed fibroblasts. After blocking of non-specific binding sites with 1% bovine serum albumin, rabbit anti-THF immune IgG was incubated with nitrocellulose strips for 2 h at room temperature. Peroxidase reaction was visualised with the use of 4-chloro-1-naphthol and $H_2O_2$ substrate. Lane 1 contains prestained molecular weight standards; lane 2 contains THF purified from Ha-MuSV transformed fibroblasts; lane 3 contains bovine serum albumin.

NaCl, was free of haemolytic activity (data not shown).

Soluble THF (34–61 μg per sample) purified from T24 and Ha-MuSV transformed fibroblast conditioned media did not contain detectable amounts of monoacylglycerol lipase, diacylglycerol lipase, lysophospholipase or phospholipase C.

*Immunological testing of THF*

Rabbit IgG anti-THF, rendered free of reactivity with BSA, demonstrated a strong band of reactivity at $M_r = 77{,}000$ with THF on Western immunoblots (Figure 3*b*). THF did not cross-react in Western immunoblots with antibodies to lymphocyte Perforin or with antibodies to tumour necrosis factor (data not shown).

### Discussion

In 1977, Zucker and Lysik reported that intact rat breast carcinoma cells were able to lyse erythrocytes and normal bone marrow erythroblasts during a 24 hour co-incubation period. Tumour-induced erythroid cytolysis occurred at 37°C, required direct contact between target and viable effector cells, was independent of DNA synthesis, and was mediated by integral plasma membrane proteins (DiStefano *et al.*, 1982). Since then, more than a dozen other spontaneous, viral and chemically transformed cancer cell lines have been shown to have the capacity to lyse erythrocytes (Lysik *et al.*, 1979; Zucker *et al.*, 1985*b*; DiStefano, 1986). Non-transformed cell lines lack haemolytic activity.

To understand better the haemolytic properties of cancer cells, we have purified a soluble tumour haemolytic factor (sTHF) from serum-free media produced by two different Harvey *ras* oncogene transformed cell lines. Both cell lines are highly malignant and kill virtually 100% of nude mice within 3 weeks of transplantation. An apparent paradox, however, is noted in measuring the haemolytic activity of intact transformed cell lines compared to their release of sTHF *in vitro*. Intact NIH-3T3 fibroblasts transformed by DNA from T-24 bladder cancer cells are not able to lyse RBCs *in vitro*. Nonetheless, T-24 transformed cells release large amounts of sTHP into 2-day conditioned media. One explanation for the lack of haemolytic activity of intact T-24 transformed fibroblasts might be the long duration of propagation of this cell line *in vitro* (2 years) which in other tumour cell lines has led to a disappearance of haemolytic activity (Zucker *et al.*, 1985*a*). In contrast, intact viral Ha-*ras* transformed cells readily lyse RBCs *in vitro*, but release sTHP of lower specific activity than T-24 transformed cells. A plausible hypothesis is that the haemolysis induced by intact cancer cells is mediated by a tumour membrane-bound haemolytic factor rather than s-THF. We have recently been able to extract a crude haemolytic factor from the membranes of both viral and T-24 *ras* transformed cells. The detergent extracted haemolytic factor differs from sTHF not only in its membrane localisation, but also in its chemical and heat stability and inhibition profile with pharmacological agents (Zucker, unpublished data).

In this study, we described a greater than 3,000-fold purification of sTHF from 2 day conditioned media produced by human T24 *ras* transformed fibroblasts and Ha-MuSV transformed cells. Incubation of purified sTHF with 5 million RBCs for 2 days resulted in the lysis of approximately 1% of the cells per μg protein (Table I).

THF is a protein of 66,000 Da (as demonstrated by SDS-PAGE), and is susceptible to digestion by trypsin. Soluble THF was identified on immunoblotting as a protein of approximately 77,000 Da. These small differences in molecular weight determinations are probably technical in nature. Purified THF is not a serine, cysteine or aspartic protease as demonstrated by the absence of inhibition by appropriate protease inhibitors. The metal chelator, EDTA, partially inhibited THF activity indicating the metal dependence of THF. However, sequential purification of THF did not lead to enrichment of metal-dependent gelatinolytic or collagenolytic activities, thus reducing the likelihood that THF is a member of the collagenase family of metalloprotease. Furthermore, THF was not inhibited by the collagenase-gelatinase inhibitor, tissue inhibitor of metalloproteases. THF lacked mono and diacylglycerol lipase, lysophospholipase or phospholipase C activity, thus ruling out this mechanism of red cell membrane disruption.

The possibility of a relationship between sTHP and cytokines produced by T lymphocytes and killer cells (Perforin) and activated monocytes (tumour necrosis factor) was explored in this report. Perforin resembles sTHP in terms of its molecular weight, heat instability, inactivation by metal chelation or serum, and requirement for cell–cell contact for activity, but differs in several important aspects (Henkart, 1985; Podack, 1986). Perforin is capable of lysing RBCs in minutes, whereas THF requires a 2 day incubation. Perforin is localised in lymphocyte granules, whereas THF is readily released by tumour cells *in vitro*. Antibodies to Perforin did not cross-react with sTHF on immunoblotting or dot blotting. Of interest, Perforin has been isolated from a long-term lymphocyte cell line that no longer has cytolytic capacity (Henkart, 1985) which is analagous to our purification of THF from T24 transformed fibroblasts that lack cytolytic activity.

Antibodies to murine tumour necrosis factor (Beutler *et al.*, 1985) did not cross-react with sTHF. Tumour necrosis factor also differs from sTHP in molecular weight and biological activity.

Having purified a protein with haemolytic capacity from tumour cells, we are presented with new questions dealing with the mechanism of action of this factor and the potential role that sTHF may play in the processes of cancer invasion or in the anaemia that accompanies disseminated cancer.

The authors would like to express their appreciation to Dr Michael Viola for providing the oncogene transformed fibroblast cell lines, to Dr Pierre Heinkart for generously providing the antisera to lymphocyte cytolytic factor, to Dr Barbara Sherry for providing the antibodies to tumour necrosis factor, and to Dr Gillian Murphy for kindly providing the purified human TIMP. The authors thank Dr Rick Singer for performing the pathologic examinations, Mr Warren McKeon for maintaining the nude mouse colony, and Mr Dean Wilkie for technical assistance. Supported by a Merit Review Grant from the Veterans Administration.

**References**

BEUTLER, B., MILSARK, I.W. & CERAMI, A.C. (1985). Passive immunization against cachectin/tumor necrosis factor protects mice from lethal effect of endotoxin. *Science*, **229**, 869.

BOHLEN, P., STEIN, S., DAIRMAN, W. & UDENFRIEND, S. (1973). Fluorometric assay of proteins in the nanogram range. *Arch. Biochem. Biophys.*, **155**, 213.

CAWSTON, T.E. & MURPHY, G. (1981). Mammalian collagenases. *Methods Enzymol.*, **80**, 711.

DEFEO, D., GONDA, M.A., YOUNG, H.A. & 4 others (1981). Analysis of two divergent rat genomic clones homologous to the transforming gene of Harvey murine sarcoma virus. *Proc. Natl Acad. Sci. USA*, **78**, 328.

DISTEFANO, J.F. (1986). Role of proteases in red blood cell target cell destruction by cells transformed by Rous sarcoma virus mutants. *Cancer Res.*, **46**, 1114.

DISTEFANO, J.F., BECK, G., LANE, B. & ZUCKER, S. (1982). Role of tumor cell membrane-bound serine proteases in tumor-induced target cytolysis. *Cancer Res.*, **42**, 207.

FAROOQUI, A.A., TAYLOR, W.A., PENDLEY, C.E., COX, J.W. & HORROCKS, L. (1984). Spectrophotometric determination of lipases, lysophospholipases, and phospholipases. *J. Lipid Res.*, **25**, 1555.

HENKART, P.A. (1985). Mechanism of lymphocyte-mediated cytolysis. *Ann. Rec. Immunol.*, **3**, 31.

LAEMMLI, U.K. (1970). Cleavage of structural proteins during the assembly of the head of bacteriophage T4. *Nature*, **227**, 680.

LYSIK, R.M., COORNETTA, K., DISTEFANO, J.F. & ZUCKER, S. (1979). Bone marrow cytolysis induced by hepatoma, teratocarcinoma, and transformed fibroblasts. *Cancer Res.*, **39**, 30.

MICHELI, F. & DONATI, M. (1903). Sulle proprietà emoliche degli estratti di organi e di tumori maligni. *La Riforma Medica*, **19**, 1037.

PANZACCHI, D.J. (1902). Sul potere emolitico dell'estratto acquoso dei tumori. *La Riforma Medica*, **18**, 592.

PODACK, E.R. (1986). Molecular mechanisms of cytolysis by complement and cytolytic lymphocytes. *J. Cell. Biochem.*, **30**, 133.

REDDY, E.P., REYNOLDS, R.K., SANTOS, E. & BARBACID, M. (1982). A point mutation is responsible for the acquisition of transforming properties by the T24 human bladder carcinoma oncogene. *Nature*, **300**, 149.

SPINUCCI, C., ZUCKER, S., WIEMAN, J.M. & 5 others (1988). Purification of a gelatin degrading type IV collagenase secreted by ras oncogene-transformed fibroblasts. *J. Natl Cancer Inst.*, **80**, 1416.

STEVEN, F.S., HULLEY, T.P. & GRIFFIN, M.M. (1982). Evidence for metal inhibition of tumour membrane-bound neutral protease and the control of tumour-induced target cell cytolysis. *Br. J. Cancer*, **46**, 934.

TRAVIS, J., BOWEN, J., TEWKSBURY, D., JOHNSON, D. & PANNELL, R. (1976). Isolation of albumin from whole human plasma and fractionation of albumin-depleted plasma. *Biochem. J.*, **157**, 301.

WEIL, R. (1907). Hemolytic properties of organ and tumor extracts. *J. Med. Res.*, **16**, 287.

WIEMAN, J., ZUCKER, S., WILKIE, D. & LYSIK, R.M. (1986). Purification of a hemolytic factor from ras oncogene transformed fibroblasts. *Biochem. Biophys. Res. Commun.*, **140**, 365.

ZUCKER, S. & LYSIK, R. (1977). Cancer-induced cytolysis of normal bone marrow cells. *Nature*, **165**, 736.

ZUCKER, S., LYSIK, R.M., RAMAMURTHY, N.S., GOLUB, L.M., WIEMAN, J.M. & WILKI, D.P. (1985*b*). Diversity of melanoma plasma membrane-proteinases: inhibition of collagenolytic and cytolytic activity by minocycline. *J. Natl Cancer Inst.*, **42**, 2705.

ZUCKER, S., LYSIK, R.M., WIEMAN, J., WILKIE, D.P. & LANE, B. (1985*a*). Diversity of human pancreatic cancer cell proteinases: role of cell membrane metalloproteinases in collagenolysis and cytolysis. *Cancer Res.*, **45**, 6168.